# A comprehensive evaluation of emotional responsiveness in borderline personality disorder: a support for hypersensitivity hypothesis

**DOI:** 10.1186/s40479-019-0105-4

**Published:** 2019-05-09

**Authors:** Roberta Bortolla, Marco Cavicchioli, Marco Galli, Paul F. M. J. Verschure, Cesare Maffei

**Affiliations:** 1 Vita-Salute San Raffaele University, via Stamira d’Ancona, 20, Milan, Italy; 2 Clinical Psychology and Psychotherapy Unit, IRCCS San Raffaele Hospital, Milan, Italy; 3 Laboratory of Synthetic Perceptive, Emotive and Cognitive Systems, Institute for Bioengineering of Catalonia (IBEC), Barcelona Institute of Science and Technology, Barcelona, Spain; 4 ICREA, Institucio Catalana de Recerca IEstudis Avançats, Passeig Lluis Companys, Barcelona, Spain

**Keywords:** Borderline personality disorder, Emotional vulnerability, Linehan’s model, Hypersensitivity, Slow return to emotional baseline

## Abstract

**Background:**

Many experimental studies have evaluated Linehan’s biological emotional vulnerability in Borderline Personality Disorder (BPD). However, some inconsistencies were observed in operationalizing and supporting its components. This study aims at clarifying which aspects of Linehan’s model are altered in BPD, considering a multimodal evaluation of processes concerned with emotional responsiveness (self-report, psychophysiology and eye-tracking).

**Methods:**

Forty-eight socio-emotional pictures were administered to 28 participants (14 BPD, 14 Healthy Controls, HCs), gender- and age-matched, by employing two different lengths of stimuli exposure (5 s and 15 s).

**Results:**

Our results supported the hypersensitivity hypothesis in terms of faster physiological responses and altered visual processing. Furthermore, hypersensitivity was associated with detailed socio-emotional contents. Hyperreactivity assumption was not experimentally sustained by physiological and self-report data. Ultimately, the slow return to emotional baseline was demonstrated as an impaired emotional modulation.

**Conclusions:**

Our data alternatively supported the hypersensitivity and the slow return to emotional baseline hypotheses, postulated by Linehan’s Biosocial model, rather than the hyperreactivity assumption. Results have been discussed in light of other BPD core psychopathological processes.

## Background

Borderline Personality Disorder (BPD) is a severe mental disorder characterized by a pervasive pattern of instability in affect regulation, impulse control, interpersonal relationships, and self-image [1]. One of the most influential theories of BPD development is Linehan’s Biosocial model [2] that posits emotional dysregulation as the core feature of such disorder. According to this model, emotional dysregulation emerges from continuous transactions between a biological emotional vulnerability and invalidating environments [3, 4]. Specifically, biological vulnerability had been conceptualized considering three different, albeit interrelated, dimensions of emotional functioning: a) heightened emotional sensitivity (e.g., low threshold for emotional reactions); b) intense emotional responses; c) slow return to emotional baseline (e.g., long-lasting emotional reactions). Given the clinical relevance of Linehan’s model for BPD treatment, several experimental studies aimed at empirically testing its dimensions, especially referring to the biological vulnerability. Despite the increasing interest on such topic, empirical findings are controversial and the operationalization of constructs largely varies across studies [5, 6].

Hypersensitivity was originally conceptualized as the tendency to pick up emotional cues (e.g., negative cues), to react quickly, and to have a low threshold for emotional reactions [2]. From an experimental perspective, the hypersensitivity hypothesis was investigated using several physiological indexes and cognitive tasks. Many authors operationalized the low threshold for emotional reactions by assuming a basal physiological hyperarousal, considering different physiological indexes, such as heart rate (HR), skin conductance response (SCR) and level (SCL) and cortisol level (for a review, see: [5]). In addition, Kuo and colleagues [7] specifically referred to basal hyperarousal considering the basal vagal tone according to Porges’s Polyvagal theory [8]. Furthermore, the heightened emotional sensitivity was assessed by evaluating the speed of facial emotion recognition (e.g., [9, 10]), assuming a proneness to rapidly perceive emotional signals in social contexts. Ultimately, emotional hypersensitivity was also investigated postulating a tendency to experience neutral stimuli characterized by an emotional valence [11, 12]. As a whole, it seems that emotional hypersensitivity is ascribed to a general proneness to react. However, no studies investigated whether physiological systems are implicated in such predisposition to emotionally react. Accordingly, an earlier onset of physiological response in relation to the presentation of emotional cues might be considered a reliable index of BPD emotional hypersensitivity. In addition, emotional hypersensitivity might be captured by attentional mechanisms. For instance, this construct was conceptualized as a condition of hypervigilance towards social stimuli [13]. Further operationalization included a faster orientation towards negative emotional faces. A meta-analysis conducted by Kaiser and colleagues [14] partially supported Linehan’s hypersensitivity hypothesis by revealing negative attentional bias for BPD schema congruent words and moderate attentional bias for positive emotional faces. Moreover, a recent work from Kaiser and colleagues [15] extended the previous findings [14] showing that BPD attentional bias was enhanced for threating information. Accordingly, BPD patients seemed mostly characterized by difficulties in disengaging attention from threating cues, rather than an initial allocation towards emotional stimuli, in line with other studies [16, 17]. Despite these results were in line with the concept of hypersensitivity, the data acquisition procedures limited to draw conclusion about the role of attentional processes in operationalizing such dimension of Linehan’s Biosocial model. Indeed, all studies mentioned above considered the manual reaction time and the error rates as outcomes of attentional dysfunctions. However, the use of these measures was called into question in studying attentional processes because they seem to evaluate late and cognitive controlled stages of attentional allocation [18]. Therefore, they do not allow to capture automatic tendencies (e.g., approach-and-avoidance) of attentional systems towards emotional cues [19]. According to the necessity to include reliable measures of attentional mechanisms, the direct estimation of visual processing patterns (e.g., eye-tracking data) represents one of the most common experimental methodologies to assess the attentional allocation on emotional cues [20–22]. Particularly, eye movements are an early, cognitively less controlled measure of attentional orienting and a biological marker of immediate shifts in visual attention [18]. Despite the wide use of eye-tracking methodology among several clinical conditions (e.g., [23, 24]) and nonclinical population [25–27], solely three studies [19, 28, 29] investigated the automatic attentional allocation on socio-emotional cues among BPD subjects. Particularly, these studies showed that BPD patients exhibited faster saccades towards the eyes of briefly presented (i.e., 150 ms) neutral faces and slower saccades away from fearful eyes [19]. Furthermore, the analysis of eye movements suggested that BPD individuals were characterized by more and faster initial fixation changes to the eyes of angry faces [28]. Eventually, Kaiser and colleagues [29] showed that BPD patients with Post-Traumatic Stress Disorder (PTSD) exhibited more and longer fixations on the eye region of angry/happy blends (ambiguous blends of facial expressions), as well as more fixations on the eye region displaying high levels of sadness compared to HCs. Bertsch and colleagues [19, 28] provided a post-hoc discussion which partially linked the evidences discussed above with Linehan’s Biosocial model. However, these results seem to marginally capture the complexity of visual processing patterns over the course of time. Indeed, according to the vigilance-avoidance hypothesis (e.g., [30]) and the eye-tracking data revealed in other clinical and non-clinical populations (e.g., [31–33]), it is possible that after an initial preference for emotional cues, BPD subjects might avoid the same cues in the later stages of visual processing. Nonetheless, no studies investigated attentional allocation patterns among BPD individuals during prolonged exposures (e.g., 5 s) to emotional stimuli in order to clarify whether emotional hypersensitivity could be reflected in visual processing mechanisms. Moreover, previous eye-tracking studies [19, 28, 29] used simple facial stimuli or ambiguous blends. To our knowledge no eye-tracking study was published on BPD sample using more complex visual stimuli.

Marsha Linehan described BPD hyperreactivity in terms of extreme reactions towards emotional stimuli [2]. BPD emotional hyperreactivity has been consistently operationalized within experimental contexts as a heightened stimulus-related change in emotional intensity (e.g., [34–36]). On the other hand, methods for assessing such aspect of Biosocial model largely varied from subjective ratings to physiological (e.g., HR, Heart Rate Variability [HRV], SCR and SCL) and behavioral (e.g., electromyography) indexes (for a review see: [37]). Given the huge amount of data concerning the experimental evaluation about this topic, a meta-analytic review was conducted [6] demonstrating that emotional hyperreactivity hypothesis was not supported with regard to several biological indexes. On the contrary, subjective ratings results showed a slightly heightened arousal in response to different emotional stimuli, as well as a tendency to negatively appraise emotional cues. Therefore, BPD emotional hyperreactivity should be mainly related to subjective experiences rather than to altered physiological responses.

The original conceptualization of Biosocial model posited the slow return to emotional baseline in terms of long-lasting physiological reactions to emotional stimuli [2]. The slow return to emotional baseline hypothesis was explicitly examined in few studies [38–40], which consistently operationalized and supported this dimension as a delayed habituation of physiological responses after the repetitive presentation of emotional cues. According to Linehan’s model [2] and its extension (i.e., Emotional Cascade Model (e.g., [41])), BPD long-lasting emotional reactions might also reflect the well-documented difficulties in using flexible emotion regulation strategies (e.g., rumination, thought suppression) across different situations [42]. This aspect could be manifested in altered HRV, given its association to a flexible modulation of physiological systems across different conditions [43, 44]. Accordingly, HRV might represent a further valid outcome for the slow return to emotional baseline hypothesis as conceptualized within the Biosocial framework. BPD patients could manifest altered HRV in response to emotional situations, as a manifestation of their inflexible emotional regulation. In addition, given their well-established impairment in habituation [38–40], this condition could be particularly manifested when they deal with prolonged emotional situations. As a consequence, a further biological conceptualization of the slow return to emotional baseline should include the evaluation of the modulation mechanisms of physiological systems in response to long-lasting emotional situation.

With regard to the three biological components, the social context seems particularly problematic for BPD patients [45]. The relevance of social stimuli for those with BPD was repeatedly confirmed by previous studies (e.g., [13, 46]). For instance, BPD patients manifested impaired emotional recognition for complex social emotional stimuli, whereas they did not show difficulties in recognizing isolated facial emotional information [46]. In addition, other authors referred to an enhanced social sensitivity reflected by increased vigilance for social stimuli, especially for cues that signaled social rejection or threat (e.g., [13]). Eventually, Weinberg and colleagues [40] showed a specific hyperreactivity to social stressor tasks, whereas no support for hyperreactivity was found considering standard stressor tasks [6].

Taking into account the previous inconsistencies in the operationalization of Linehan’s Biosocial model dimensions and considering the inconclusive empirical findings related to BPD emotional responsiveness, this study aimed at experimentally investigating the emotional hypersensitivity and hyperreactivity hypotheses and the slow return to emotional baseline assumption. Self-report, physiological and eye-tracking data were collected to test the dimensions mentioned above. In line with conceptual considerations discussed previously, the Biosocial dimensions were operationalized considering different indexes of emotional functioning as following:

- With regard to physiological markers, it was assumed that BPD emotional hypersensitivity was effectively conceptualized by an earlier onset of skin conductance response (reduced latency of skin conductance response [SCR Latency]) or/and by heightened basal arousal (increased HR, SCR and SCL; reduced Root Mean Square of the Successive Differences [RMSSD]). Referring to eye-tracking data, it was hypothesized that BPD hypersensitivity could influence both spatial and temporal dimensions of visual processing patterns of emotional cues. In particular, considering a prolonged exposure to complex emotional stimuli, the current study assumed that BPD individuals might exhibit an overall avoidance of socio-emotional contents in terms of reduced spatial and temporal visual exploration (reduced gazes and time spent in Areas of Interest [AoIs]).
- Emotional hyperreactivity was operationalized by enhanced stimulus-related changes in emotional intensity, considering both physiological and self-report indexes. According to meta-analytic results [6], no significant differences would be expected between BPD subjects and healthy controls (HCs) considering physiological indexes (HR, SCR, SCL, RMSSD). On the contrary, a higher self-report arousal might be observed among BPD individuals.
- The slow return to emotional baseline was conceptualized as a difficulty in adapting emotional responses to prolonged emotional situations. Specifically, physiological and self-report results were compared between two experimental conditions, which differed from each other in terms of exposition time to emotional stimuli (i.e., 5 s and 15 s). This choice was supported by studies reporting significant effects of stimuli exposition time on processing mechanism among BPD patients [47, 48]. In line with Fenske and colleagues’ findings [48], it was postulated that BPD subjects might show increasing difficulties in emotion modulation, both considering physiological (RMSSD) and self-report indexes (arousal, valence and dominance) during prolonged exposure to emotional stimuli.

Ultimately, given the well-established difficulties of BPD individuals with social interaction (e.g., [45]), this study employed complex daily-life social pictures in order to improve the naturalistic approach of the experiment. Furthermore, two different types of AoIs were used to analyze eye-tracking data. Particularly, it was taken into consideration either AoI strictly and explicitly connected to emotion expression in social contexts, either larger AoI which included other features in addition to the previous elements. This choice was proposed with the aim of demonstrating that attentional mechanisms associated to BPD emotional hypersensitivity should be specifically related to socio-emotional cues.

## Methods

### Participants

#### BPD patients

Fourteen BPD outpatients were included from the Clinical Psychology and Psychotherapy Unit of San-Raffaele Hospital (Milan) from January to April 2017. This sample was composed by 11 female and 3 male subjects (mean age = 28.42, *SD* = 7.43). With regard to educational background, 10 patients (71.4%) had high school degree, 2 (14.3%) had a bachelor degree, and 2 (14.3%) had higher University degree (M.Sc.). Average years of education was 14.00 (*SD* = 1.71). Clinical subjects met a BPD diagnosis according to DSM-IV criteria assessed by the *Structured Clinical Interview for DSM-IV axis II Personality Disorders, Version 2.0* (SCID-II) [49]. SCID-II was conducted during the routine diagnostic assessment by trained raters, who were blinded to the hypotheses of this study. The number of BPD traits ranged from 5 to 9 (M = 6.17, SD = 1.75). Exclusion criteria were represented by IQ lower than 70, psychotic disorders and other active psychiatric symptomatology for at least 1 month before task administration (e.g. *major* depressive episode, current substance use, panic attacks). Lifetime co-diagnoses of other psychiatric disorders did not represent exclusion criteria from the study. Expert psychiatrists conducted clinical interviews for evaluating the presence of exclusion criteria. The mean number of Personality Disorders (PDs) diagnoses was 1.53 (*SD* = 0.52, range 1–2). PD co-diagnoses were Narcissistic PD (*N* = 2, 14.3%), Histrionic PD (*N* = 2, 14.3%), Passive-Aggressive PD (*N* = 3, 21.4%) and Depressive PD (*N* = 1, 7.14%). Eight patients (57.14%) did not report any psychiatric comorbidity. Furthermore, 4 patients presented a lifetime psychiatric disorder in comorbidity, namely Major Depressive Disorder (*N* = 1, 7.14%), Eating Disorder (*N* = 2, 14.29%), and Substance Use Disorders (*N* = 1, 7.14%). Pharmacological treatments did not represent exclusion criteria from the study, according to meta-analytic results [6] that showed no relevant confounding effects of this clinical aspect on BPD physiological responsiveness. Thirteen patients took stable pharmacological treatments for at least 3 months. The number of medical prescriptions ranged from 2 to 3. The most commonly prescribed medications were SSRIs (*N* = 5, 35.71%), anticonvulsant (*N* = 4, 28.57%), antiepileptics (*N* = 4, 28.57) and neuroleptics (typical and atypical, *N* = 4; 14,28%), benzodiazepines (*N* = 7, 50.0%) and antidepressant (*N* = 3, 21.43%). However, patients were asked to refrain from taking benzodiazepines 48 h before the experiment.

#### Healthy controls

Fourteen community dwelling volunteers with negative medical history for psychiatric or neurological disorders were included in the nonclinical sample. HCs were 10 female and 4 male subjects (mean age = 27.57, SD = 5.9). With regard to educational background, 7 participants (50.0%) had high school degree, 5 (35.7%) had a bachelor degree, and 2 (14.3%) had higher University degree (M.Sc.). Mean educational level was 14.79 years (S.D. = 1.97). No significant differences in educational level were found comparing BPD and HC groups (U = 76.50, *ns*). Participants were preliminarily screened for investigating the presence of certificated psychological, psychiatric and neurological diagnoses, as well as their related treatments. Additional exclusion criteria were IQ lower than 70, current substance use, psychopharmacological treatments and current or lifetime psychological treatments. Ultimately, the Personality Inventory for DSM-5 (PID-5) [50] and the Difficulties in Emotion Regulation Scale (DERS) [51] were administered in order to exclude the presence of maladaptive personality features and emotional difficulties.

### Instruments

#### Pictures

Forty-eight pictures from the Nencki Affective Picture System (NAPS) [52] were administered during the experiment. One-hundred and sixty-one stimuli were selected by the authors of the study from the entire NAPS database including pictures which clearly represented human emotional expressions and social interactions within daily life contexts. The set was administered to 120 community dwelling volunteers who rated each picture on two continuous bipolar semantic sliding scales (arousal and valence) each ranging from 1 to 9, following the procedure of the original database validation [52]. Pictures with mean valence ratings less than 4 were classified as negative; those between 4 and 6 were classified as neutral, and those with mean valence ratings higher than 6 were classified as positive. Sixteen pictures for each valence category (positive, negative, neutral) were selected considering the stimuli that were the most representative for that category. Mean valence was 7.31 (*SD* = .73) for positive picture, 2.64 (*SD* = .77) for negative pictures and 4.56 (*SD* = .62) for neutral pictures.

Pictures were divided in two blocks with different presentation lengths: *long* (15 s) and *short* (5 s). Five-seconds picture presentation corresponds to the guidelines for EDA data processing [53]. Moreover, the 15 s exposure was chosen to support a reliable assessment of HRV indexes [54]. No significant differences between blocks were found considering valence and arousal ratings for positive (valence: t_(14)_ = 1.10, *ns*; arousal: t_(14)_ = − 1.29, *ns*), negative (valence: t_(14)_ = − 1.29, *ns*; arousal: t_(14)_ = .23, *ns*) and neutral pictures (valence: t_(14)_ = −.86, *ns*; arousal: t_(14)_ = .83, *ns*). Inter-trial intervals between pictures varied from 10 to 15 s. The order of the stimuli presentation was randomized in order to avoid possible carry-over effects. Selected pictures are listed in Table 1.

**Table 1:** List of selected pictures

Short block			Long block		
Positive Pictures	Negative Pictures	Neutral Pictures	Positive Pictures	Negative Pictures	Neutral Pictures
Faces_064	Faces_011	Faces_037	Faces_101	Faces_021	Faces_006
Faces_089	Faces_032	Faces_154	Faces_129	Faces_158	Faces_039
Faces_092	Faces_145	Faces_162	Faces_130	Faces_173	Faces_060
Faces_107	Faces_283	Faces_204	Faces_184	Faces_174	Faces_144
Faces_127	Faces_296	Faces_206	Faces_258	Faces_285	Faces_264
Faces_232	People_127	Faces_213	Faces_358	Faces_293	Faces_281
Faces_240	People_136	People_060	People_048	People_085	Faces_289
People_192	People_137	People_139	People_176	People_126	Faces_301

#### Physiological data

EDA and ECG data were collected by using *BITalino* [55, 56]. *BITalino* is a biomedical data acquisition device with a sampling rate of 1000 Hz. EDA was collected by the employment of two electrodes on the left palm. EDA results were examined through Ledalab (www.ledalab.de). A 2 Hz low-pass filter was employed for pre-processing the data [57]. Moreover, a *Continuous Decomposition Analysis* [58] was applied and the principal skin conductance indexes were detached in a 5 s response window after the presentation of each picture. Tonic (SCL), Phasic (SCR) Skin Conductance Response, as well as SCR Latency were calculated from raw physiological data. To normalize the data, a log transformation was used. ECG data was processed by using Kubios (https://www.kubios.com/). Artifact correction and smoothness priors methodology were applied. Heart rate (HR) and the square root of the mean squared differences of successive NN intervals (RMSSD) were calculated from raw ECG data. SCL, SCR and HR were consistent with the literature regarding emotional reactivity evaluation, while RMSSD was considered as an effective and reliable index of physiological adaptation. Lastly, SCR Latency was calculated as an operationalization of Linehan’s physiological hypersensitivity.

#### Eye-tracking data

Eye-tracking data were collected by using *The Eye-Tribe* (www.theeyetribe.com), and numerous temporal and spatial outcomes were extracted with Python 2.7. Two types of Areas of Interest (AoI) were designed. First of all, experimental AoIs (AoIs) were a priori defined by the authors, selecting the part/s of the picture which explicitly contained relevant social contents (e.g., emotional faces, social interactions). On the contrary, pilot AoIs (AoIp) were defined by a preliminary study on a non-clinical sample (*N* = 12) including the portion of the picture mostly looked by the participants on the base of the application of a Gaussian filter on the data. Ultimately, AoIs and AoIp shared the same part of the picture although they varied in their size. In detail, AoIp contained other elements in addition to explicit social contents (e.g., hands, legs, whole body). *Prop gaze* (i.e., the ratio of the participant’s number of gazes in AoI and the total number of gazes for the picture), *mean time* (i.e., the participant’s mean time spent in AoI), *time 1st fixation* (i.e., the amount of time required for reaching the first AoI) and *1st fixation duration* (i.e., the amount of time spent in the first looked AoI) were calculated for each picture for AoIs and AoIp. When the picture enclosed more than one AoI, we summed the eyes values for each AoI, with the exclusion of *mean time* for which the mean values were calculated. Each visual index was corrected by the size of the AoI, excluding *time 1st fixation* which was corrected by the distance among AoI and the position of the first gaze. Finally, delta scores were calculated for each visual outcome subtracting AoIs from AoIp values, for effectively comparing the visual indexes in the previous areas. Positive scores indicated a predominant orientation of attention on the elements of AoIp that were not included in AoIs. Conversely, negative scores indicated a main focus on detailed socio-emotional contents.

#### Self-report

Arousal, valence and dominance were rated on a continuous scale ranging from 0 to 1 using three digital sliders (Affective Sliders, AS) [59]. In agreement with the authors of the AS, the dominance slider was added to the two original scales (arousal and valence). The dominance dimension indicated changes in emotional control in response to a specific emotional cue. Lower scores denoted higher control over emotional activation, whereas higher scores represented the feelings of being overwhelmed by the emotional activation elicited by the picture. The poles of the AS (Aroused/Relaxed; Positive/Negative; Dominant/Overwhelmed) were categorized with the presence of an emoticon (i.e., symbolic and stylized facial expression) to give a visual representation of the affective poles of the scales.

##### Positive and negative affect schedule (PANAS) [60]

The PANAS is a 20-item questionnaire developed for assessing the current positive (Positive Affect, PA) and negative (Negative Affect, NA) affectivity. The PANAS is constituted by 10 positive and 10 negative adjectives. Subjects were asked to rate on a 5-point Likert scale (from 1= “*very slightly or not at all*” to 5 = “*extremely*”) how much they felt as indicated by the 20 adjectives (e.g., active, determined, excited, nervous, scared, distressed, etc.). The sum of the 10 positive and negative adjectives was calculated to provide a total score for PA and NA, respectively. The original validation showed that the two scales were highly internally consistent, largely uncorrelated, and stable at appropriate levels over a 2-month time period [60]. In our sample, we administered the Italian version of the scale [61]. The PANAS factor structure and solid psychometric proprieties were also confirmed for the Italian version [61].

##### Difficulties in emotion regulation scale (DERS) [51]

The DERS is a 36-items multidimensional questionnaire for evaluating six emotion dysregulation scales (Nonacceptance of Emotional Responses; Difficulties Engaging in Goal-Directed Behavior; Impulse Control Difficulties; Lack of Emotional Awareness; Limited Access to Emotion Regulation Strategies; and Lack of Emotional Clarity). Participants were asked to rate the 36 items on a 5-point Likert scale ranging from 1 (“*almost never*”) to 5 (“*almost always*”). DERS total score was given by the sum of the 36 items. Moreover, scale scores were calculated for the six subscales. Robust psychometric proprieties were reported for the original version with an internal consistency of .93, a test-retest reliability of .88 during a 4-week to 8-week interval, and a clear factor structure. Additionally, good construct validity and a high internal consistency was found in clinical and nonclinical populations [51]. The Italian version of the instrument was administered in our sample [62]. The DERS factorial structure, good internal consistency and test-retest reliability were replicated for the Italian version [62].

##### Personality inventory for DSM-5 (PID-5) [50]

The PID-5 is a 220-item questionnaire evaluating DSM-5 maladaptive personality traits and domains. The 220 items were rated on a 4-point Likert scale from 0 (“*very false or often false”*) to 3 (“*very true or often true*”). The PID-5 has 25 primary scales that load onto 5 higher order dimensions (Negative affectivity, Detachment, Disinhibition, Antagonism and Psychoticism). As a result of differences in scale length, mean scale scores were used instead of sum, both for the 5 domains and the 25 subscales. Adequate internal consistency was found for PID-5 traits and domains [50]. In our sample, the Italian version of the questionnaire was administered [63]. Robust psychometric proprieties were confirmed also for clinical and nonclinical Italian samples, both with regard to internal consistency and factorial structure [63].

### Procedure

The complete process was carried out in laboratory setting at San-Raffaele Hospital, Milan from 11.00 a.m. to 2.00 p.m. Participants were asked to refrain from drinking coffee 2 h before the experiment or smoking cigarettes 1 h before the experiment. Additionally, alcohol or illicit drugs used 24 h before the experiment represented an exclusion criterion. Informed consent was signed prior to the experiment. Participants completed the PANAS and other pre-task questionnaires (e.g., additional clinical and medical information). A 2 min baseline for physiological parameters was recorded. Then, participants were asked to complete a small practice part of 5 pictures. The experiment was composed by two blocks of 24 pictures each (*long* and *short* setup), with a small break between them. The order of the stimuli presentation was randomized for each participant, as well as the order of the blocks. Before each picture, a fixation cross was displayed for 1 s on the screen and participants were instructed to look at the cross until the picture appeared. Pictures were presented for 5 s or 15 s, accordingly to the block. Subsequently, three rating scales (Arousal, Valence and Dominance) were presented on the screen. Subjects were instructed to rate each scale using the mouse and then to move to the next picture by clicking on a “*Continue*” button on the screen. During the whole procedure, EDA, ECG and eye-tracking data were continuously recorded. After the task, participants completed the PANAS. The DERS and the PID-5 were completed by participants after the experiment, at home.

### Statistical analysis

Non-parametric procedures were proposed to analyze data in line with the small sample size and the violations of normal distribution for several measures included in the study. Particularly, Mann-Whitney *U* test was computed to compare BPD and HC subjects considering clinical variables assessing the severity of BPD psychopathology (i.e., DERS and PID-5 scores) and state variables associated to the experimental context (i.e., pre- and post-task PANAS scores). The exact test was used to compute *p*-values. The *r* coefficient was calculated as an effect size measure for non-parametric comparisons between groups [64, 65]. The Aligned Rank Transform using ARTool program [66] was applied in order to evaluate non-parametric main effect of group and interaction effects group by valence of emotional stimuli (i.e., negative, positive, neutral; *group x category*) and block (i.e., short and long; *group x block*), taking into account indexes of emotional functioning measured during the task (i.e., self-report, physiological and eye-tracking data). The aligned transformation refers to a preprocessing procedure that aligns the data for each effect (main or interaction) before assigning ranks averaged in the case of ties. Data alignment is an established process in statistics [67] by which effects are estimated as marginal means and then “stripped” from the response variable so that all effects but one are removed. After the aligned rank transformation of data for each effect, factorial ANOVA was conducted to evaluate the significance of main and interaction effects, which was estimated using the *F-*test [66]. Partial *η*^2^ (_*p*_*η*^*2*^) was utilized as an effect size measure of non-parametric main and interaction effects. Post-hoc comparisons were based on Mann-Whitney *U* tests using exact test for the estimation of *p*-values. With regard to post-hoc analyses, adequate Bonferroni’s correction was applied when multiple comparisons were performed. Ultimately, Monte Carlo simulation based on 10,000 independent samples and its 2-*tailed* 99% confidence interval (CI) was employed in order to evaluate the robustness of between-group comparisons. Spearman’s correlation was used to evaluate associations among different emotional response systems (i.e., self-report scores, physiology and eye-tracking).

## Results

Table 2 shows between group comparisons concerning emotional state variables (i.e., PANAS pre- and post-task scores) and questionnaires scores (i.e., DERS and PID-5 scores). As expected, BPD subjects reported significantly higher scores in all PID-5 domains/traits and DERS scales compared to HCs, with the exception of DERS Awareness subscale and PID-5 Antagonism domain and Risk Taking facet. The analyses did not detect significant differences in pre-task PANAS scores. Therefore, it was possible to exclude confounding effects of pre-existing emotional state on responsiveness to experimental paradigm. Furthermore, no significant differences were observed in PANAS post-task levels and pre- post-task change scores (PANAS PA: *U* = 80.50, *Z* = −.82, *ns*; PANAS NA: *U* = 85.50, *Z* = −.58, *ns*).

**Table 2:** Descriptive statistics and non-parametric comparisons related to questionnaires data

		BPD (*N* = 14)		HCs (*N* = 14)		*U*	*Z*	*r*
		M (SD)	Median	M (SD)	Median			
DERS	DERS TOT	3.01 (.69)	3.19	2.05 (.60)	1.91	26.00	3.155^	.60
	Clarity	13.53 (5.07)	16.00	9.57 (3.52)	9.50	46.55	2.17*	.41
	Non Acceptance	18.53 (6.66)	20.00	11.71 (4.87)	11.00	34.50	2.75**	.51
	Goals	17.38 (5.11)	19.00	12.21 (3.91)	11.00	32.50	2.85^	.54
	Impulse	17.76 (5.39)	17.00	11.35 (5.18)	10.00	34.00	2.77**	.52
	Awareness	15.07 (4.79)	16.00	13.71 (4.44)	14.50	76.00	.731	.14
	Strategies	26.00 (8.15)	28.00	15.28 (6.01)	12	27.00	3.11^	.59
PID-5 BPD *facets*	Anxiousness	2.33 (.48)	2.33	.84 (.55)	.89	4.50	4.21^^	.79
	Emotional Lability	2.52 (.44)	2.72	1.00 (.61)	.93	5.50	4.16^^	.79
	Hostility	1.70 (.72)	1.80	.66 (.47)	.70	23.50	3.28**	.62
	Separation Insecurity	1.67 (.59)	1.57	.49 (.31)	.57	9.50	3.97^^	.75
	Depressivity	1.96 (.70)	2.07	.24 (.25)	.14	2.50	4.30^^	.81
	Risk Taking	1.61 (.67)	1.86	1.21 (.59)	1.03	58.00	1.60	.30
	Impulsivity	1.70 (.81)	1.67	.81 (.57)	.75	34.00	2.78^^	.52
PID-5 *domains*	Negative Affectivity	1.97 (.30)	1.98	.96 (.24)	.93	1.00	4.28^^	.19
	Detachment	1.48 (.54)	1.49	.43 (.24)	.38	10.00	3.82^^	.72
	Antagonism	.91 (.44)	.90	.55 (.50)	.49	38.00	2.38	.45
	Disinhibition	1.46 (.42)	1.37	.99 (.34)	.97	32.00	2.69*	.51
	Psychoticism	1.35 (.47)	1.31	.41 (.43)	.10	12.00	3.72^^	.70
PANAS *pre-task*	PA	28.64 (6.44)	27.50	26.00 (4.24)	25.00	75.50	1.00	.19
	NA	29.00 (6.52)	27.00	25.9 3 (3.20)	25.50	74.50	1.10	.21
PANAS *post-task*	PA	25.71 (7.30)	26.00	24.07 (5.59)	22.50	86.50	.529	.10
	NA	26.36 (6.36)	24.50	24.78 (4.11)	24.50	89.50	.392	.07

*DERS* Difficulties in emotion regulation scale, *PID-5* Personality inventory for DSM-5, *PANAS* Positive and negative affect schedule, *PA* Positive affectivity, *NA* Negative affectivity

*p < .05; ** *p* < .01; ^ *p* < .005; ^^ *p* < .001

Tables 3, 4 and 5 summarize descriptive statistics concerning self-report, eye-tracking and physiological data collected during the task. Table 6 summarizes factorial ANOVA results for self-report, physiological and visual indexes.

**Table 3:** Descriptive statistics related to self-reported data

	Condition	BPD (*N* = 14)		Total M (SD)	HCs (*N* = 14)		Total M (SD)
		*Long* M (SD)	*Short* M (SD)		*Long* M (SD)	*Short* M (SD)	
Valence	Negative	.27 (.13)	.26 (.13)	.27 (.12)	.30 (.12)	.20 (.11)	.25 (.11)
	Positive	.61 (.12)	.68 (.17)	.64 (.15)	.65 (.09)	.71 (.13)	.68 (.11)
	Neutral	.41 (.13)	.42 (.13)	.42 (.12)	.41 (.07)	.37 (.09)	.40 (.07)
	Total	.43 (.08)	.46 (.08)	.46 (.06)	.46 (.03)	.43 (.03)	.47 (.03)
Arousal	Negative	.52 (.14)	.51 (.19)	.52 (.15)	.43 (.23)	.54 (.24)	.48 (.23)
	Positive	.42 (.19)	.51 (.21)	.47 (.19)	.37 (.23)	.42 (.23)	.39 (.22)
	Neutral	.43 (.13)	.45 (.18)	.44 (.14)	.36 (.20)	.39 (.19)	.37 (.19)
	Total	.49 (.15)	.49 (.15)	.49 (.14)	.38 (.21)	.45 (.19)	.44 (20)
Dominance	Negative	.38 (.24)	.37 (.25)	.38 (.24)	.29 (.26)	.36 (.26)	.33 (.26)
	Positive	.23 (.17)	.25 (.18)	.24 (.17)	.25 (.22)	.29 (23)	.27 (.22)
	Neutral	.27 (.18)	.23 (.18)	.25 (.16)	.23 (.21)	.25 (.22)	.24 (.21)
	Total	.29 (.19)	.28 (.19)	.31 (.19)	.26 (.22)	.30 (.21)	.30 (.22)

**Table 4:** Descriptive statistics related to eye-tracking data in experimental and pilot Areas of Interest

	Condition	BPD (*N* = 14)		Total M (SD)	HCs (*N* = 14)		Total M (SD)
		*Long* M (SD)	*Short* M (SD)		*Long* M (SD)	*Short* M (SD)	
Prop gaze (AoIs)	Negative	.005 (.001)	.006 (.001)	.006 (.001)	.007 (.001)	.006 (.001)	.006 (.001)
	Positive	.008 (.002)^c^	.008 (.002)^b^	.008 (.002)^a*^	.010 (.002)^c^	.010 (.002)^b^	.010 (.002)^a*^
	Neutral	.008 (.002)	.006 (.001)^b^	.007 (.002)	.009 (.002)	.007 (.001)^b^	.008 (.002)
	Total	.007 (.002)	.007 (.001)^b^	.007 (.001)^a*^	.009 (.002)	.008 (.001)^b^	.008 (.001)^a*^
time 1st fix (AoIs)	Negative	.051 (.031)	.321 (.729)	.180 (.344)	.050 (.035)	.039 (.044)	.045 (.026)
	Positive	.052 (.068)	.129 (.236)^b^	.090 (.149)	.044 (.062)	.032 (.014)^b^	.038 (.030)
	Neutral	.064 (.103)	.073 (.056)	.069 (.070)	.024 (0.15)	.165 (.331)	.094 (.166)
	Total	.056 (.045)	.171 (.303)	.135 (.229)	.039 (.022)	.079 (.109)	.041 (.020)
1st fix duration (AoIs)	Negative	.007 (.003)	.007 (.003)	.007 (.003)	.008 (.003)	.009 (.003)	.009 (.002)
	Positive	.007 (.001)	.007 (.003)^b^	.00 7(.002)	.009 (.004)	.009 (.003)^b^	.009 (.003)
	Neutral	.010 (.006)	.006 (.003)^b^	.008 (.004)	.012 (.005)	.009 (.004)^b^	.011 (.003)
	Total	.008 (.003)	.007 (.003)^b*^	.007 (.002)^a*^	.010 (.003)	.009 (.002)^b*^	.009 (.002)^a*^
Mean time (AoIs)	Negative	.007 (.002)	.005 (.002)	.006 (.002)	.009 (.003)	.006 (.002)	.007 (.002)
	Positive	.009 (.003)^c^	.007 (.002)	.008 (.002)^a^	.012 (.004)^c^	.009 (.004)	.011 (.003)^a^
	Neutral	.011 (.004)	.005 (.004)	.008 (.004)	.014 (.006)	.007 (.002)	.010 (.004)
	Total	.009 (.003)^c^	.006 (.003)^b^	.007 (.002)^a*^	.012 (.003)^c^	.007 (.002)^b^	.009 (.002)^a*^
Prop gaze (AoIp)	Negative	.004 (.001)	.004 (.001)^b^	.004 (.001)^a^	.004 (.001)	.005 (.001)^b^	.004 (.001)^a^
	Positive	.004 (.001)	.003 (.000)	.004 (.001)	.005 (.001)	.003 (.000)	.004 (.000)
	Neutral	.005 (.001)	.005 (.001)	.005 (.001)	.006 (.001)	.005 (.001)	.006 (.001)
	Total	.004 (.001)	.004 (.001)	.004 (.000)^a^	.005 (.001)	.005 (.001)	.004 (.001)^a^
time 1st fix (AoIp)	Negative	.088 (.161)	.016 (.014)	.052 (.081)	.050 (.067)	.018 (.026)	.034 (.033)
	Positive	.031 (.047)	.017 (.023)	.024 (.026)	.038 (.089)	.011 (.020)	.025 (.044)
	Neutral	.119 (.314)	.015 (.014)	.067 (.156)	.027 (.029)	.025 (.031)	.026 (.020)
	Total	.038 (.041)	.016 (.012)	.038 (.041)	.038 (.035)	.018 (.015)	.029 (.027)
1st fix duration (AoIp)	Negative	.006 (.003)	.071 (.246)^b^	.038 (.123)	.007 (.004)	.007 (.002)^b^	.007 (.002)
	Positive	.005 (.002)^c^	.005 (.002)^b*^	.005 (.001)	.007 (.005)^c^	.008 (.003)^b*^	.008 (.003)
	Neutral	.016 (.039)	.016 (.039)^b^	.013 (.021)	.010 (.007)	.009 (.003)^b^	.010 (.004)
	Total	.030 (.083)	.031 (.083)^b*^	.022 (.062)^a*^	.008 (.005)	.008 (.002)^b*^	.007 (.003)^a*^
Mean time (AoIp)	Negative	.005 (.002)	.004 (.002)	.005 (.002)	.006 (.002)	.005 (.003)	.006 (.002)
	Positive	.006 (.002)	.004 (.002)	.005 (.001)	.008 (.003)	.004 (.003)	.006 (.002)
	Neutral	.007 (.002)^c^	.006 (.004)	.007 (.002)	.012 (.002)^c^	.005 (.002)	.009 (.004)
	Total	.006 (.002)	.005 (.002)	.005 (.001)	.009 (.004)	.005 (.002)	.006 (.002)

*AoIs*: Experimental areas of interest, *AoIp*: Pilot areas of interest

Note: Significant differences between groups are marked by identical superscripted letters (*p* < .05)

*significant after Bonferroni correction

**Table 5:** Descriptive statistics related to physiological data

	Condition	BPD (*N* = 14)		Total M (SD)	HCs (*N* = 14)		Total M (SD)
		*Long* M (SD)	*Short* M (SD)		*Long* M (SD)	*Short* M (SD)	
SCR	Negative	1.69 (.89)	1.93 (.89)	1.73 (.55)	1.86 (.48)	1.86 (.38)	1.86 (.40)
	Positive	1.81 (.60)	2.02 (1.13)	1.83 (.62)	1.97 (.50)	1.85 (.42)	1.91 (.40)
	Neutral	1.88 (.50)	1.84 (.80)	1.83 (.50)	1.84 (.49)	1.79 (.35)	1.82 (.37)
	Total	1.79 (.54)	1.92 (.89)	1.78 (.58)	1.90 (.44)	1.83 (.33)	1.88 (.38)
SCL	Negative	823.90 (83.02)	824.90 (93.78)	818.33 (87.15)	845.60 (180.96)	806.59 (128.80)	833.19 (135.81)
	Positive	841.35 (58.76)	806.15 (122.39)	817.39 (95.60)	794.08 (126.93)	804.69 (143.76)	799.23 (132.91)
	Neutral	817.98 (91.90)	800.96 (115.90)	811.32 (97.57)	797.93 (123.15)	821.82 (106.28)	809.85 (113.88)
	Total	822.57 (84.60)	801.03 (119.25)	817.60 (91.75)	816.33 (118.25)	810.46 (125.62)	817.90 (116.60)
Latency	Negative	1.49 (.11)	1.62 (.19)	1.55 (.09)^a*^	1.68 (.22)	1.68 (.20)	1.68 (.14)^a*^
	Positive	1.49 (.11)	1.63 (.42)	1.55 (.19)	1.65 (.24)	1.59 (.18)	1.61 (.17)
	Neutral	1.61 (.23)	1.50 (.12)	1.55 (.15)^a^	1.70 (.29)	1.71 (.30)	1.70 (.21)^a^
	Total	1.53 (.10)^c^	1.59 (.22)	1.55 (.13)	1.68 (.18)^c^	1.66 (.18)	1.65 (.14)
HR	Negative	77.55 (9.46)	78.47 (10.91)	78.09 (9.94)	76.71 (13.18)	76.99 (12.42)	78.82 (12.71)
	Positive	77.11 (10.50)	78.80 (9.47)	77.61 (9.73)	75.47 (13.49)	77.08 (12.53)	76.26 (12.99)
	Neutral	76.93 (10.30)	77.91 (11.36)	77.28 (10.97)	75.24 (10.66)	76.98 (11.99)	76.06 (11.22)
	Total	77.21 (9.90)	78.08 (10.55)	77.86 (9.81)	75.80 (12.18)	77.01 (12.18)	76.55 (12.81)
RMSSD	Negative	40.44 (15.66)	40.47 (30.54)	38.76 (15.97)	42.27 (16.61)	35.11 (16.44)	38.81 (14.70)
	Positive	37.45 (17.04)	33.39 (12.71)	36.06 (13.86)	41.90 (13.04)	32.94 (12.87)	37.44 (12.23)
	Neutral	39.02 (17.69)	41.27 (27.46)	38.82 (18.81)	47.89 (14.23)	32.11 (14.55)	40.13 (12.92)
	Total	38.96 (15.53)	39.41 (25.43)	37.51 (14.41)	44.02 (13.20)	33.38 (12.70)	38.13 (12.92)

*SCR:* Skin conductance response, *SCL:* Skin conductance level, *RMSSD:* Root mean square successive difference

Note: BPD sample was composed by 9 subjects when SCR, SCL and Latency outcomes were considered

Significant differences between groups are marked by identical superscripted letters (*p* < .05)

*significant after Bonferroni correction

**Table 6:** Factorial ANOVA results. *Group* effect and *Group x Category* and *Group x Block* interactions for included indexes

Variable	*Group F (1,26)*	*Group*_*p*_ *ɳ* ^*2*^	*Group x category F (2,25)*	*Group x category*_*p*_ *ɳ* ^*2*^	*Group x Block F (2,25)*	*Group x Block*_*p*_ *ɳ* ^*2*^
Self-report						
Valence	.02	.00	.47	.63	13.54**	.34
Arousal	.28	.01	.31	.02	.25	.01
Dominance	.05	.00	.94	.07	3.05	.11
Eye-tracking						
Prop gaze (AoIs)	5.73*	.18	5.21*	.29	.02	.00
Time 1st fix (AoIs)	2.58	.09	.39	.03	.15	.01
1st fix duration (AoIs)	7.64*	.23	1.07	.08	2.67	.09
Mean Time (AoIs)	5.40*	.17	.35	.03	1.85	.07
Physiology						
SCL^a^	.07	.01	.19	.03	3.29	.17
SCR^a^	.49	.02	.07	.01	.37	.02
Latency^a^	4.39*	.17	.47	.05	.20	.01
HR	.61	.02	.44	.03	.12	.01
RMSSD	.05	.01	.39	.03	10.05^	.29

*p < .05; ** *p* < .01; ^ *p* < .005

^a^Degrees of freedom for F-test are (1,21) for Group effect and (2,20) for two ways interactions

Five BPD subjects were omitted from EDA analysis because of technical problems in recording electrodermal activity. However, we compared BPD included and excluded subjects (BPD patients with EDA recording vs. BPD patients without EDA recording) with regard to relevant clinical variables. BPD excluded subjects did not differ from included participants, considering all the clinical variables assessed in the study, namely number of BPD (*U* = 15.5, *ns*) and other PDs’ traits (*all p < .05*), DERS (*U* = 37.0, *ns*) and PID-5 scores (*U*_*neg. aff*_ = 9.0, *ns; U*_*detach*_ = 14.0, *ns; U*_*antag*_ = 9.0, *ns; U*_*disin*_ = 15.0, *ns; U*_*psychot*_ = 13.0, *ns*), number of psychiatric disorders (*U* = 7.0, *ns*) and pharmacological treatment (number and type of prescriptions; *all p < .05*).

### Hypersensitivity

The analyses did not reveal between-group differences considering basal physiological responses (SCR, SCL, HR and RMSSD). On the contrary, a significant main effect of group was found for SCR Latency (*F*(1,21) = 4.39; *p* < .05, _*p*_*η*^*2*^ = .17). Specifically, BPD patients exhibited a faster onset of physiological responses (see Table 5), mostly in relation to the exposure to negative (*U* = 24.00; *Z* = − 2.46; *p* < .0167, Monte Carlo 99% CI: [.009–.015]; *r* = −.46) and neutral (*U* = 32.00, *Z* = − 1.95; *p* = .05; Monte Carlo 99% CI: [.04–.06]; *r* = −.36) stimuli. Exclusively the first comparison is significant after Bonferroni correction (α/3 = .0167).

As regard to eye-tracking data, a significant main effect of group was found for *prop gaze* (*F*(1,26) = 5.73; *p* < .05, _*p*_*η*^*2*^ = .18). Particularly, BPD patients showed a significant lower number of gazes when exploring socio-emotional cues included in AoIs (see Table 4), especially in relation to stimuli characterized by a positive valence (*U* = 46.00; *Z* = − 2.39; *p* < .0167, Monte Carlo 99% CI: [.01–.016]; *r* = −.45) (Bonferroni correction: α/3 = .0167). A significant main effect of group was also observed with respect to *1st fixation duration* (*F*(1,26) = 7.64; *p <* .05; _*p*_*η*^*2*^ = .23). Specifically, BPD subjects spent significantly less time in the first looked AoIs (see Table 4) compared to HCs. Ultimately, the analyses revealed a significant main group effect for the *mean time* (*F*(1,26) = 5.40; *p <* .05; _*p*_*η*^*2*^ = .17) spent in AoIs, with BPD patients reporting shorter mean time in AoIs compared to HCs. Overall, BPD patients spent less time in exploring socio-emotional cues as manifested by lower *(prop) gaze*, shorter *1st fixation duration* and shorter *mean time* compared to HCs (see Table 4). On the contrary, no significant between-group difference was found for *time 1st fixation*.

### Hyperreactivity

The analyses did not find significant main and interaction effects in relation to the size of stimulus-related changes in self-report (i.e., arousal, valence, dominance) and physiological (i.e., SCR, SCL, HR, RMSSD) measures.

### Slow return to emotional baseline: difficulties with emotion modulation

**Fig. 1:**
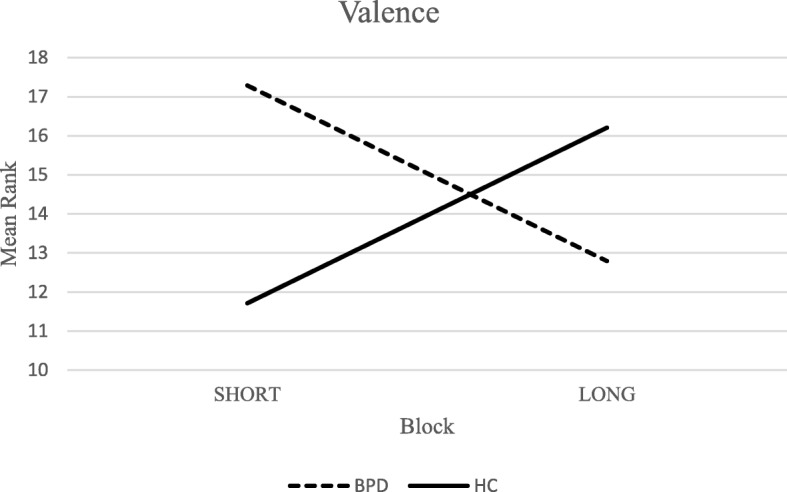
Group x Block interaction for Valence ratings

**Fig. 2:**
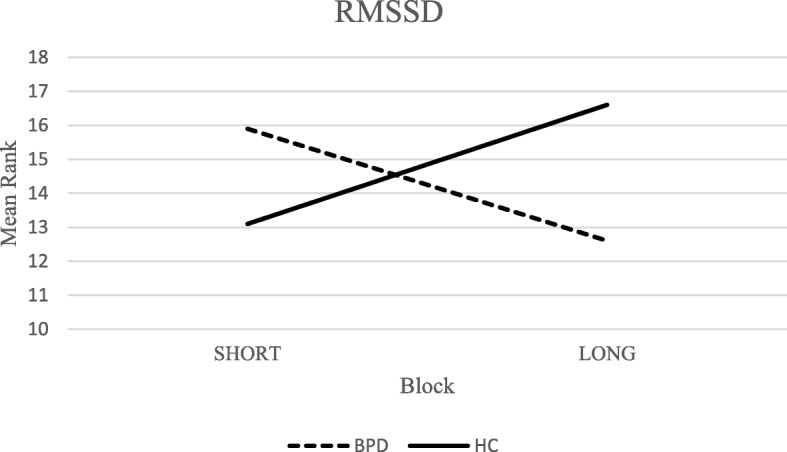
Group x Block interaction for RMSSD

A *group x block* interaction was observed for valence ratings (*F*(2,25) = 13.54; *p* < .01; _*p*_*η*^*2*^ = .34) (Fig. 1). Particularly, BPD patients reported a reduction of valence ratings when results from the short block (5 s) were compared to the long one (15 s). On the contrary, HCs exhibited an opposite pattern of self-report responses. The difference between these trends was significant (*U* = 27.50; *Z* = − 3.24; *p <* .01; Monte Carlo 99% CI: [.000–.002]; *r* = −.61) (Bonferroni correction: α/2 = .025). No further interaction effects were detected for the other subjective experience domains. Considering the physiological indexes, the analyses showed a significant *group x block* interaction for RMSSD (*F*(2,20) = 10.05; *p* < .01; _*p*_*η*^*2*^ = .29) (Fig. 2). The RMSSD was higher for stimuli presented for 5 s, compared to 15-s presentation condition, among BPD subjects. Conversely, HCs exhibited higher levels of RMSSD when they dealt with stimuli presented for 15 s. The difference between blocks for RMSSD levels was significant comparing BPD subjects with HCs (*U =* 35.00, *Z* = -2.89, *p* < .01; Monte Carlo 99% CI: [.002–.005]; *r* = −.54) (Bonferroni correction: α/2 = .025).

### The effect of socio-emotional content

When pilot AoI were considered, the significant main group effect was replicated exclusively for *1st fixation duration* (*F*(1,26) = 8.09; *p <* .01; _*p*_*η*^*2*^ = .24). With respect to this eye-tracking index, it was also observed a significant *group x category* interaction effect (*F*(2,25) = 3.53; *p* < .05; _*p*_*η*^*2*^ = .22). Specifically, the duration of the 1st fixation was significantly shorter among BPD subjects compared to HCs, when considering stimuli characterized by a positive valence (*U* = 39.00; *Z* = − 2.71; *p* < .0167; Monte Carlo 99% CI: [.002–.006]; *r =* −.51) (Bonferroni correction: α/3 = .0167).

Computing delta scores (i.e., pilot – experimental) of eye-tracking indexes between AoIp and AoIs, significant differences between groups were detected considering *prop gaze* (*U* = 53.00; *Z* = 2.07; *p* < .05; Monte Carlo 99% CI: [.015–.022]; *r =* .39) and *mean time* (*U* = 55.00; *Z* = 1.98; *p* < .05; Monte Carlo 99% CI: [.020–.028]; *r =* .37). Particularly, BPD group showed larger differences in visual processing pattern between AoIp and AoIs than HCs.

### Congruency among self-report, physiological and eye-tracking measures

No significant correlation was found among self-report, physiological and eye-tracking indexes in BPD patients and in HCs.

## Discussion

This study sought to investigate the dimensions that characterize BPD biological emotional vulnerability, as hypothesized by Linehan’s Biosocial model [2]. Particularly, it was adopted a comprehensive assessment approach (i.e., self-report, physiological, eye-tracking) in order to test and to propose an alternative operationalization of the hypersensitivity and the hyperreactivity hypothesis, as well as considering the slow return to emotion baseline assumption. Ultimately, it was also explored how selective socio-emotional cues could influence visual processing patterns of BPD individuals, taking into account the well-established difficulties with social interactions associated to such disorder [45].

Our results partially supported the hypersensitivity hypothesis as demonstrated by an earlier onset of sympathetic response to social cues. This proneness to physiologically react seems to be particularly relevant for stimuli characterized by a negative and neutral valence. According to these findings, the original conceptualization of emotional hypersensitivity, which described it as a reduced threshold for emotional responses, might be explained by a tendency to rapidly react to different kind of emotional cues. On the one hand, considering the effect of negative emotional stimuli on the onset of physiological response, this finding might be in accordance with other studies [68–70] demonstrating that BPD subjects showed a specific alteration in physiological responsivity to negative emotional situations. On the other hand, the rapid physiological response associated to the presentation of stimuli characterized by a neutral valence might reflect the well-documented tendency of BPD subjects to negatively interpret neutral cues (for a review see: [12]). Furthermore, the hypersensitivity assumption seems to be supported by altered visual processing mechanisms in exploring emotional stimuli. As expected, spatial and temporal eye-tracking indexes suggested that BPD subjects are characterized by a reduced visual exploration of socio-emotional cues. This finding partially replicated Bertsch and colleagues’ results [28] showing that BPD subjects exhibited alterations in visual exploration when dealing with selective emotional cues (e.g., eyes). Furthermore, according to the vigilance-avoidance hypothesis (e.g., [30]) and other eye-tracking studies (e.g., [31–33]), BPD subjects might massively use attentional avoidance strategies [71], especially when they deal with explicit social-emotional contents. This aspect of BPD functioning could be ascribed to the well-established association to rejection sensitivity [72] that leads BPD patients to misinterpret social signals as untrustworthy and avoiding them as a consequence [73–75]. Interestingly, eye-tracking data revealed a lower number of gazes in AoIs when BPD subjects were exposed to stimuli characterized by a positive valence. This finding could be in line with theoretical and empirical evidence that demonstrated that BPD subjects tend to suppress the positive elements of internal experiences and external situations in the light of cognitive schemas (for a review, see: [76]) that filter incongruent information with self-others dysfunctional representations [77, 78]. Nonetheless, future experimental studies should be carried out in order to validate the hypothesized relationship between rejection sensitivity and dysfunctional cognitive representations with attentional and visual processes.

Considering no significant differences in the amplitude of physiological responses and in self-report levels of emotional reactivity (i.e., arousal, valence, dominance), the hyperreactivity hypothesis was not supported by the current experimental task. This result is not surprising in the light of meta-analytic conclusions [6] that showed consistent and null effect sizes between BPD subjects and HCs, taking into account several physiological indexes of emotional reactivity. On the contrary, different trends between BPD subjects and HCs were observed in self-report and cardiac responses when the length of stimuli presentation was considered. Specifically, BPD subjects rated the longer stimuli (15 s) as more negative than the ones presented for 5 s. Furthermore, the clinical group showed lower levels of RMSSD index in the 15-s exposure condition than in the other condition. Conversely, HCs showed inverse trends considering both indexes. Taken these findings together, it could be possible to conclude that BPD subjects, contrary to HCs, exhibited difficulties in adapting physiological systems implicated in emotion regulation [43, 44, 79, 80] when dealing with prolonged exposure to socio-emotional situations. This kind of physiological alteration might preliminary explain the biological underpinnings that contribute to the conceptualization of the slow return to emotional baseline. Strictly connected to this biological aspect and the results regarding self-report levels of negative valance, the slow return to emotional baseline might be also attributed to the maladaptive effects of the rigid use of emotion regulation strategies (e.g., rumination, experiential avoidance). It was previously demonstrated that maladaptive emotion regulation affects both emotional subjective experience [41, 42] and long-term physiological responses [81–84]. Eventually, this aspect could be heightened when BPD subjects are exposed to long-lasting emotional situations. Nevertheless, future research should empirically investigate how dysfunctional emotion regulation strategies are involved in explaining the slow return to emotional baseline operationalized as an overall difficulty in adapting physiological systems to contextual variability.

Referring to visual processing mechanisms implicated in defining emotional hypersensitivity, the results supported that specific socio-emotional cues should be considered the core challenging aspects of a situation for BPD subjects. Indeed, the current findings suggested that BPD patients seem to avoid facial expressions and interpersonal cues in favor of an orientation of attention to other elements of the picture. Accordingly, the well-established BPD difficulties in managing social interactions could be ascribed to an incapability to appraise complex socio-emotional signals.

Ultimately, no significant associations were found considering self-report, physiological and eye-tracking responses to socio-emotional pictures in BPD patients and HCs. This finding is in line with Cavazzi and Becerra’s review [5] and with the results of a current meta-analysis by Bortolla, Cavicchioli and colleagues [6] supporting the hypothesis of a substantial decoupling among subjective experience, physiological activation, and behavioral responses in BPD patients. From the perspective of the Emotional Coherence theory [85], different components of emotional response (i.e., subjective response, behavior and physiology) are rarely coherent, and they respond in multiple ways to environmental demands especially in psychopathology. As a consequence, treatments for BPD need to incorporate gradual exposure to specific emotionally provocative stimuli to enhance the emotional coherence between the different emotional systems. However, further research is needed to deepen this topic.

Despite the considerations mentioned above, several limitations need to be discussed. First of all, although an adequate statistical methodology was applied to support the robustness of findings and the number of participants were similar to other psychophysiological studies on BPD emotional responsiveness [35, 85, 86], the sample size represents the primary limitation to the results’ generalizability, especially considering electrodermal data. Therefore, future replication studies administering the current experimental task should be carried out in order to sustain the operationalization of emotional hypersensitivity and the slow return to emotional baseline proposed in the current study. Moreover, the small sample did not allow to control the results for possible confounding effects of gender on emotional responsiveness, as demonstrated in other neuroimaging (e.g., [87]) and psychophysiological (e.g., [88, 89]) studies. Thus, further studies should test BPD gender differences in emotional dysregulation, taking into account several domains of emotional functioning (i.e., self-report, physiological, attentional).

Secondly, although our findings on eye-tracking indexes were in accordance with the hypothesis of altered attentional processes, it is possible that the reduced BPD visual exploration could be explained by secondary elements of the pictures that attract their focus of attention. Thus, these results should be replicated by comparing social cues with non-social pictures (e.g., objects, landscapes). Furthermore, given the high co-occurrence of other psychiatric disorders (e.g., major depressive disorder, anxiety disorders) among BPD subjects and the absence of clinical control groups characterized by overlapped physiological and attentional dysfunctions [90–92], it was not possible to definitively ascribed emotional dysfunctions observed in the current study to BPD pathology. As a result, future research investigating the biological emotional vulnerability of Linehan’s Biosocial model should compare BPD subjects with other psychopathological conditions to clarify the core dysfunctional mechanisms associated to the disorder.

## Conclusion

In conclusion, this is the first study that evaluated subjective, physiological and visual processing implicated in the comprehensive operationalization of the biological emotional vulnerabilities postulated by the Linehan’s Biosocial model. The results supported the hypersensitivity and the slow return to emotional baseline hypotheses. On the contrary, the hyperreactivity hypothesis was not sustained by empirical data.
